# Supplementary material for: Active particles bound by information flows
Source: Nat Commun. 2018 Sep 21;9:3864. doi: 10.1038/s41467-018-06445-1 (PMC6154969; doi:10.1038/s41467-018-06445-1)
Supplement: Supplementary file 1 — Supplementary Information [file 41467_2018_6445_MOESM1_ESM.pdf]

Supplementary Information - Active Particles Bound by Information Flows

Utsab Khadka[1], Viktor Holubec[2,3], Haw Yang[1], Frank Cichos[4*]

[1] *Department of Chemistry, Princeton University, Princeton, New Jersey 08544, USA.*
[2] *Institute for Theoretical Physics, Universität Leipzig, 04103 Leipzig, Germany.*
[3] *Charles University, Faculty of Mathematics and Physics, Department of Macromolecular Physics, V Holešovičkách 2, CZ-180 00 Praha, Czech Republic.*
[4] *Peter Debye Institute for Soft Matter Physics, Universität Leipzig, 04103 Leipzig, Germany. E-mail: cichos@physik.uni-leipzig.de*

Supplementary Note 1: Symmetric Active Particle Velocity

The active particle used throughout the experiments is symmetric in structure, with 30 % of the melamine resin particle surface covered with gold nanoparticles (AuNP). The propulsion velocity v_{th} is the result of an asymmetric illumination with a highly focused laser at a wavelength of $\lambda = 532\,\mathrm{nm}$, which heats the gold nanoparticles at the surface and thus creates a surface temperature gradient and corresponding thermo-osmotic creep flows. A sketch of the particle and a corresponding electron microscopy image is shown in Supplementary Figure 1.

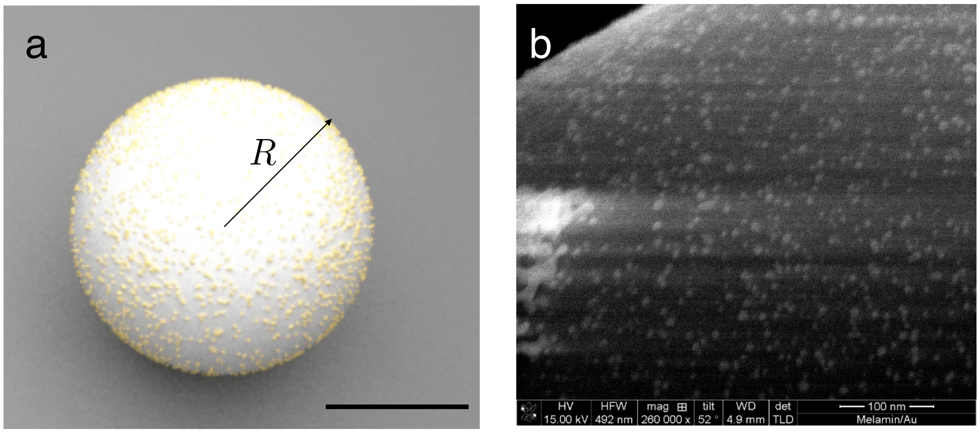

Supplementary Figure 1 | (**a**) Sketch of the symmetric active particle of radius R. For the experiments a melamine particle of $R = 1.09\,\mu\mathrm{m}$ covered with 10 nm gold nanoparticles at 30 % of its surface is heated with a focused laser. The scale bar corresponds to $1\,\mu\mathrm{m}$.(**b**) Electron microscopy image of the gold nanoparticle at the surface of the melamine resin particle (kindly provided by Santiago Muiños Landin, Molecular Nanophotonics Group).

Active particle velocity and laser focus displacement. As explained in the main text, the magnitude and the direction of the expected active particle velocity depends on the displacement of the heating laser focus from the center of the particle. The displacement of the laser focus from the particle center is also responsible for the nonlinear power dependence of the propulsion velocity. To model this nonlinear dependence, we simplify the 3-dimensional geometry and only consider the 2-dimensional situation sketched in Supplementary Figure 2. The z-direction denotes the direction perpendicular to the sample plane, while the x-direction lies in the sample plane. For heating the gold nanoparticles at the surface of the active particle, we assume a Gaussian heating beam with an intensity profile

$$I(x) = I_0 \exp(-(x - \delta x)^2 / 2\omega_0^2). \tag{1}$$

Here ω_0 is the beam radius and the center of the Gaussian is displaced by δx with respect to the particle center as indicated in Supplementary Figure 2. The beam radius is assumed to be constant in the sample region and thus independent of the z-position. To obtain an expression for the propulsion velocity as a function of the laser displacement

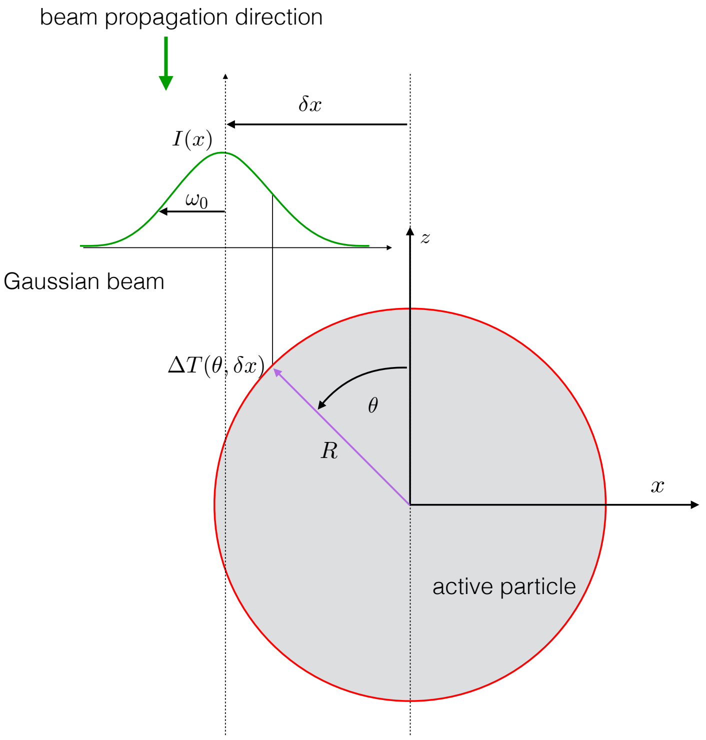

Supplementary Figure 2 | Definition of symbols for modeling the nonlinear power dependence of the active particle velocity. An one dimensional intensity profile $I(x)$ representing a Gaussian beam with a beam waist of ω_0 is assumed to propagate along the z-direction. The beam center is displaced by δx from the center of a symmetric swimmer of radius R. To calculate the resulting propulsion velocity we consider the intensity at an angle θ on the particle surface.

δx, we need to determine the temperature profile along the particle surface, i.e., the circle circumference in the described model geometry. To do so we project the Gaussian intensity profile to the circle of radius R by substituting $x = -R\sin(\theta)$ into supplementary equation 1. Assuming that the temperature increment due to the heating is proportional to the incident laser intensity, $\Delta T(\theta, \delta x) \propto I(\theta, \delta x)$ we obtain the temperature increase as compared to the ambient temperature T_0 along the circumference of the circle as

$$\Delta T(\theta, \delta x) = T(\theta, \delta x) - T_0 = \Delta T_0 \exp(-(R\sin(\theta) - \delta x)^2/2\omega_0^2). \quad (2)$$

ΔT_0 indicates the maximum temperature increase at the particle circumference. The tangential temperature gradient along the circumference is then

$$\nabla_{||} T = \frac{\partial \Delta T(\theta, \delta x)}{\partial \theta} = -\Delta T(\theta, \delta x) \frac{R\cos(\theta)(R\sin(\theta) - \delta x)}{\omega_0^2}. \quad (3)$$

This tangential temperature gradient leads to a quasi thermo-osmotic slip velocity $v_{\mathrm{s}} = \chi \nabla_{||} T$ setting the hydrodynamic boundary condition at the particle surface (with the thermo-osmotic mobility coefficient χ)[1,2] . The propulsion velocity v_{th} of the active particle is then

the surface average (circumference average here) of the slip velocity vector (supplementary equation 4). For symmetry reasons all components perpendicular to the x-axis cancel out and only the components along the x-direction represented by the additional $\cos(\theta)$ factor need to be considered. The propulsion velocity along the x-axis is therefore

$$v_{\mathrm{th}}(\delta x) = \frac{\chi}{2\pi} \oint_{\theta} \Delta T(\theta, \delta x) \frac{R\cos^2(\theta)(R\sin(\theta) - \delta x)}{\omega_0^2} \mathrm{d}\theta. \tag{4}$$

To obtain the dependence of the particle velocity on the laser displacement δx, we numerically integrate supplementary equation 4. Supplementary Figure 3 shows the obtained particle velocity as a function of the beam displacement δx for two different beam radii ω_0 in the left panel. The right panel is indicating the laser displacement for the maximum particle velocity as a function of the particle radius. Accordingly, to obtain the maximum velocity the laser has to be placed at about a distance corresponding to the particle radius.

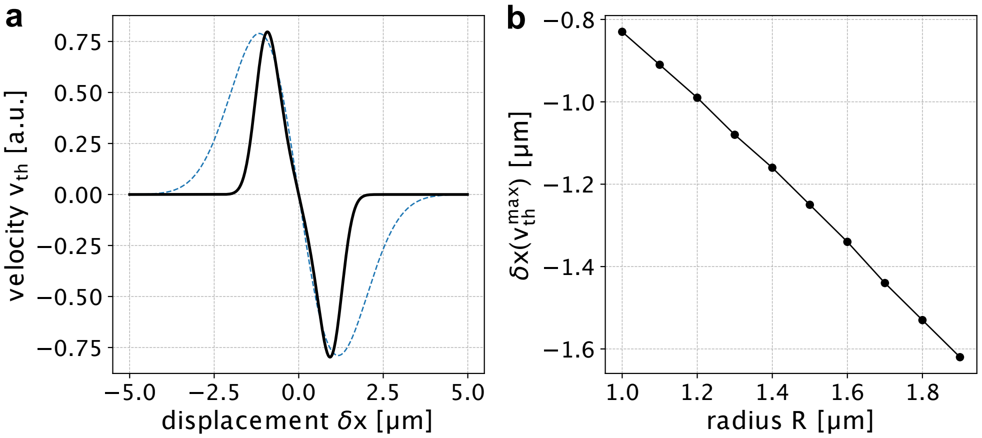

Supplementary Figure 3 | (**a**) Calculated active particle propulsion velocity as a function of the displacement δx of a Gaussian heating laser beam from the center of the particle according to supplementary equation 4 for $R = 1.09\,\mu$m and a beam radius of $\omega_0 = 0.3\,\mu$m (solid line) and $\omega_0 = 1\,\mu$m (dashed). (**b**) Dependence of the displacement δx from the particle center for a maximum velocity $v_{\mathrm{th}}^{\mathrm{max}}$ as a function of the particle radius R.

Power dependence of the active particle velocity The nonlinear power dependence of the velocity of the active particle is the result of the dependence of the velocity on the laser displacement δx and the finite exposure time Δt_{exp}. Within the exposure time, the laser is placed at the particle rim to cause the maximum velocity according to the previous section. The particle starts to move and the instantaneous velocity drops as the laser is spatially fixed. The average velocity observed is then

$$< v_{\mathrm{th}} >= \frac{1}{\Delta t_{\mathrm{exp}}} \int_{0}^{\Delta t_{\mathrm{exp}}} v_{th}(\delta x(t)) \mathrm{d}t \tag{5}$$

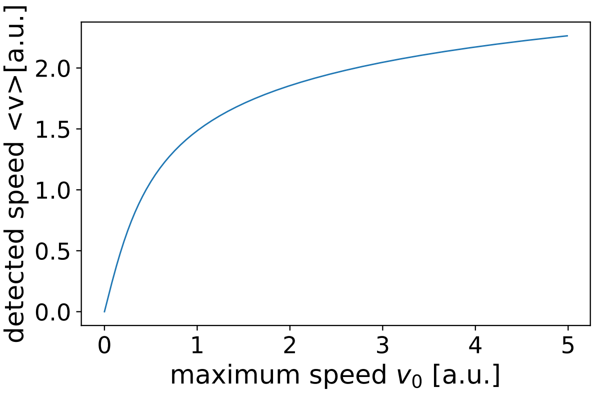

Supplementary Figure 4 | Numerical solution of supplementary equation 7 showing the average speed as a function of the maximum speed (proportional to the incident heating power) for $\omega_0 = 1\,\mu$m and $\delta x = 1\,\mu$m. The dependence captures the nonlinear increase of the detected active particle velocity as a function of the incident heating power. The nonlinearity is due to the particle moving out the laser beam during the exposure time.

The displacement δx increases with the instantaneous velocity $v_{\mathrm{th}}(\delta x)$ of the particle itself. We assume the following approximate function for the velocity

$$v_{\mathrm{th}}(\delta x) = -v_0(P)\,\frac{\delta x}{[\mu\mathrm{m}]} \cdot e^{-\frac{\delta x^2}{2\sigma^2}} \tag{6}$$

with a velocity amplitude $v_0 \propto P_{\mathrm{heat}}$, which depends linearly on the incident heating power P_{heat}, a width σ and $[\mu\mathrm{m}]$ denoting that the unit of δx is removed. The total displacement within a time period Δt_{exp} is then obtained from integrating $\mathrm{d}\delta x = v_{\mathrm{th}}(\delta x)\,\mathrm{d}t$ resulting in

$$\int\limits_{t=0}^{\Delta t_{\mathrm{exp}}} dt = \Delta t_{\mathrm{exp}} = -\int\limits_{\delta x}^{\delta x+\Delta x} \frac{[\mu\mathrm{m}]}{v_0 \delta x'} e^{\frac{\delta x'^2}{2\sigma^2}}\, d\delta x' = -\frac{[\mu\mathrm{m}]\left\{\mathrm{Ei}\left(\frac{(\delta\mathrm{x}+\Delta\mathrm{x})^2}{2\sigma^2}\right) - \mathrm{Ei}\left(\frac{\delta\mathrm{x}^2}{2\sigma^2}\right)\right\}}{2v_0} = \frac{\Delta x'}{< v_{th} >}. \tag{7}$$

Supplementary equation 7 has to be solved to determine the total displacement $\Delta x'$ of the active particle during the time period Δt_{exp} and thus for the average velocity. A solution is only available by numerical integration. The obtained velocity as a function of the maximum velocity v_0 is displayed in Supplementary Figure 4 and captures the trend observed experimentally. Note that in the case of multiple particles being heated, the exposure time has to be replaced by the heating time $\Delta t_{\mathrm{heat}} = \Delta t_{\mathrm{exp}}/N$ (N is the total number of particles interacting), which is due to the multiplexing of the heating beam. A more detailed description of the properties of the symmetric active particles will be published elsewhere.

Active particle motion The experimental results shown in Figure 1 b–d of the main text have been obtained from a driving of the particle back and forth between two target positions (see Supplementary Fig. 5a) and a localization for 100 frames at each of the target positions.

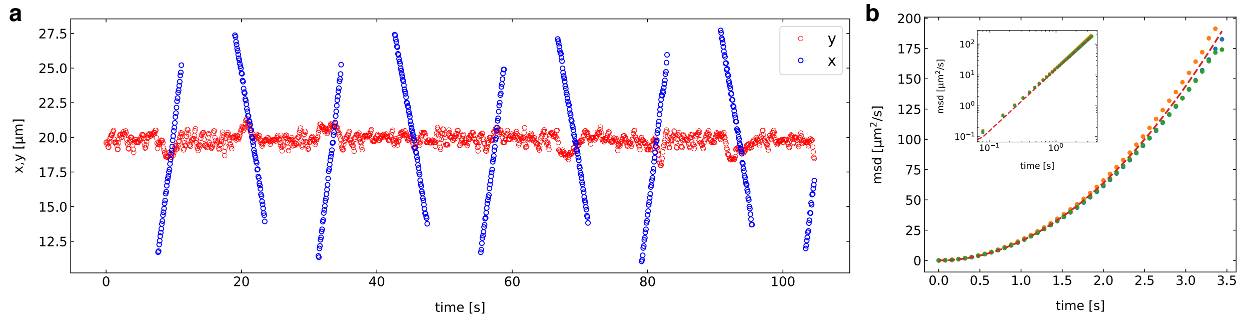

Supplementary Figure 5 | (**a**) Positions (x, y) of a single active particle driven between two target locations along the x-direction as presented in Figure 1b of the main text. The incident laser power is $P = 1\,\mathrm{mW}$. (**b**) Mean squared displacement (msd) calculated from the driving periods of the experiments shown in (a). The graph displays the msd (inset: double log scale) for three different driving periods as a function of time for a single particle at an incident laser power of $P = 1\,\mathrm{mW}$ as well as the predicted parabolic time dependence according to the measured velocity of $v_{\mathrm{th}} = 3.9\,\mu\mathrm{m\,s^{-1}}$ (dashed line).

As the rotational diffusion of the particle is not influencing the motion, the mean squared displacement during these driving periods should be purely parabolic. Supplementary Figure 5 shows the mean squared displacement during three different driving periods taken from the experiments shown in Figure 1 b–d of the main text. The particle is propelled with an incident laser power of $P = 1\,\mathrm{mW}$. The resulting velocity is $v_{\mathrm{th}} = 3.9\,\mu\mathrm{m\,s^{-1}}$. The experimental mean squared displacement is purely parabolic over the time period of 3.5 seconds and agrees well with the expected one for a driven motion with constant velocity (dashed line).

Supplementary Note 2: Simulated Structures

Brownian simulations of the active particle molecules have been carried out to obtain comparative snapshots of the formed structures (Figure 3 main text) not aiming at a quantitative comparison. Simulations use the Processing environment (http://www.processing.org). For each particle we define a velocity and a noise amplitude to take care of the propulsion and the Brownian motion of the particles. The speed of the active particle has been set to 6 pixel/frame. Gaussian distributed noise with a variance of 1 pixel has been added to the particle positions at each frame. The propulsion direction is set according to the rules defined in the main text with a defined value of r_{eq} corresponding to 80 pixels. The feedback delay is set to one frame, meaning that the positions in the previous frame are used to calculate the directions of propulsion. Similar to the experiments, particle self-organize into a dynamic structure from which snapshots are taken and depicted in Figure 3 of the main text.

Supplementary Note 3: Analytical Description of the Particle Dynamics

Dimer bond length oscillation To evaluate the active particle molecule dynamics we consider the special case of a dimer. For two active particles at the positions $\mathbf{r}_1(t)$ and $\mathbf{r}_2(t)$ we may write down two Langevin equations in the overdamped limit to study their dynamics,

$$\begin{aligned} \dot{\mathbf{r}}_1(t) &= \mathbf{v}_1(t) + \sqrt{2D_0}\eta_1, \quad (8) \\ \dot{\mathbf{r}}_2(t) &= -\mathbf{v}_1(t) + \sqrt{2D_0}\eta_2. \quad (9) \end{aligned}$$

The two equations can be combined to give the dynamics of the bond vector $\mathbf{r}_{12}(t) = \mathbf{r}_1(t) - \mathbf{r}_2(t)$:

$$\dot{\mathbf{r}}_{12}(t) = 2\mathbf{v}_1(t) + \sqrt{4D_0}\eta_{12} = -2v_{\text{th}}\text{sign}\left(|\mathbf{r}_{12}(t-\delta t)| - r_{\text{eq}}\right)\mathbf{e}_{12}(t-\delta t) + \sqrt{4D_0}\eta_{12}, \quad (10)$$

where η_{12} is a zero-mean, unit-variance Gaussian white noise (vector), i.e., $(\langle\eta_{12}(t)\rangle = 0$ and $\langle\eta(t)\eta(t')\rangle = \delta(t-t'))$ such that the variance of the noise term in supplementary equation 10 corresponds to $4D_0$. The velocity $|\mathbf{v}_1| = v_{\text{th}}$ is the propulsion velocity and δt the feedback delay time. Note that for $\delta t \neq 0$ it is not guaranteed that the vectors $\mathbf{r}_{12}(t) = r_{12}\mathbf{e}_{12}(t)$ and $\mathbf{e}_{12}(t-\delta t)$ are parallel. For vanishing thermal noise ($D_0 = 0$), however, the situation simplifies because then the vectors $\mathbf{r}_{12}(t) = r_{12}\mathbf{e}_{12}(t)$ and $\mathbf{e}_{12}(t-\delta t)$ are always parallel and the motion of the dimer becomes effectively one-dimensional. Setting $D_0 = 0$ in supplementary equation 10 and taking the scalar product of the result and $\mathbf{e}_{12}(t) = \mathbf{e}_{12}(t-\delta t)$ we obtain the formula

$$\dot{r}_{12}(t) = -2v_{\text{th}}\text{sign}\left(|r_{12}(t-\delta t)| - r_{\text{eq}}\right). \quad (11)$$

This equation can be solved with the result

$$r_{12}(t) = r_{\text{eq}} + Ax_{\text{tr}}(t+\phi_0), \quad (12)$$

where $A = 2v_{\text{th}}\delta t$ is the amplitude of oscillations and

$$x_{\text{tr}}(t) = \frac{8}{\pi^2}\sum_{k=0}^{\infty}(-1)^k\frac{\sin\left(2\pi(2k+1)ft\right)}{(2k+1)^2} \quad (13)$$

denotes a triangular wave with amplitude 1 and period $T = 1/f = 4\delta t$. The phase shift ϕ_0 is determined by the initial condition which is assumed to be drawn from the interval $[r_{\text{eq}} - A, r_{\text{eq}} + A]$ attained by the solution 12 in the stationary state.

Scalar multiplication of supplementary equation 10 by the vector $\mathbf{e}_{12}(t)$ yields

$$\dot{r}_{12}(t) = -2v_{\text{th}}\text{sign}\left(|r_{12}(t-\delta t)| - r_{\text{eq}}\right)\cos\alpha + \sqrt{4D_0}\eta_{12}, \quad (14)$$

where $\cos\alpha = \cos\alpha(t, t-\delta t) = \mathbf{e}_{12}(t)\cdot\mathbf{e}_{12}(t-\delta t)$. For experimentally relevant parameters (the distance diffused in the direction perpendicular to $\mathbf{e}_{12}(t-\delta t)$ per δt is small compared to the minimal inter-particle distance $r_{\text{eq}} - A$), it is reasonable to assume that $\cos\alpha \approx 1$. Then we arrive at the formula

$$\dot{r}_{12}(t) = -2v_{\text{th}}\text{sign}\left(|r_{12}(t-\delta t)| - r_{\text{eq}}\right) + \sqrt{4D_0}\eta_{12}. \quad (15)$$

Numerical analysis of this equation reveals that the nonzero noise leads to damped averaged oscillations of the bond. Although the exact analytical calculation of the damping constant seems to be beyond our reach due to the non-analytical nature of the force, an approximate solution can be found.

Time correlation function for the bond length The normalized time correlation function $C(t)$ of the dimer bond length fluctuations $\Delta r_{12}(t) = r_{12}(t) - r_{\mathrm{eq}}$ is defined as

$$C(t) = \frac{\int\limits_0^{n/f} \mathrm{d}\tau\, \Delta r_{12}(\tau)\Delta r_{12}(\tau + t)}{\int\limits_0^{n/f} \mathrm{d}\tau\, \Delta r_{12}^2(\tau)}. \tag{16}$$

Here, the time integration runs over n periods of the oscillations with frequency f measured in the experiment. To obtain an approximate time dependence of the correlation function including the effect of dephasing due to the Brownian motion of the particles we investigate the influence of the noise on the first term of the series 13. In this approximation the bond length is given by

$$\Delta r_{12}(t) = 8A/\pi^2 \sin(2\pi f t + \phi(t)), \tag{17}$$

where the effect of the noise is summarized in $\phi(t)$ and solely caused by thermal fluctuations of the dimer (amplitude A fluctuations are neglected). We assume that $\phi(t = 0) = 0$ and thus the wave without noise evolves according to $\Delta r_{12}^{D_0=0}(t) = 8A/\pi^2 \sin(2\pi f t)$. The distance $\Delta x(t)$ between the noise free and and the noisy solution within a time t is then given by

$$\Delta x(t) = \Delta r_{12}^{D_0=0}(t) - \Delta r_{12}(t) = \frac{8A}{\pi^2}\left[\sin(2\pi f t) - \sin(2\pi f t + \phi(t))\right]. \tag{18}$$

Assuming that $\phi(t)$ is small, one can expand the right-hand side with the result

$$\Delta x(t) = \Delta r_{12}^{D_0=0}(t) - \Delta r_{12}(t) \approx \frac{8A}{\pi^2}\left[-\cos(2\pi f t)\phi(t) + \sin(2\pi f t)\frac{\phi(t)^2}{2} + \ldots\right]. \tag{19}$$

Using the first order term we obtain

$$\phi(t) \approx \frac{\pi^2 \Delta x(t)}{8A} \tag{20}$$

assuming that the maximum phase shift is given when $|\cos(2\pi f t)| = 1$. If the displacements $\Delta x(t)$ obey a Gaussian distribution

$$p(\Delta x) = \frac{1}{\sqrt{4\pi D \Delta t}} e^{-\frac{\Delta x^2}{4D\Delta t}} \tag{21}$$

with $D = 2D_0$ due to the relative motion, where D_0 is the diffusion coefficient of a free individual active particle, one can calculate the approximate damping constant as follows.

Inserting supplementary equation 17 into the time-correlation function 16 and assuming additivity of the phase shift $\phi(t + \tau) = \phi(t) + \phi(\tau)$ gives $C(t) = \cos(2\pi f t + \phi(t))$. Averaging this expression over the ensemble of Brownian displacements 21 yields

$$C(t) = \int\limits_{-\infty}^{\infty} p(\phi)\cos(2\pi f t + \phi)\mathrm{d}\phi = \frac{\pi^2}{8A}\int\limits_{-\infty}^{\infty} p(\Delta x)\cos\left(2\pi f t + \frac{\pi^2 \Delta x}{8A}\right)\mathrm{d}\Delta x. \tag{22}$$

The oscillations with frequency f and phase shift ϕ sum up to form an exponentially decaying oscillation

$$C(t) = e^{-\pi^4 D\, t/64A^2} \cos(2\pi f t). \tag{23}$$

The time constant of the exponential decay is therefore $T_2 = 64A^2/\pi^4 2D_0$. It is named T_2 due to its analogy with the dephasing time in optical spectroscopy or magnetic resonance. Inserting the amplitude A yields

$$T_2 = \frac{256 v_{\mathrm{th}}^2 \delta t^2}{2\pi^4 D_0}. \tag{24}$$

The dephasing time is therefore decreasing with increasing noise, i.e., increasing diffusion coefficient D_0.

Supplementary Note 4: Entropy Fluxes

Without the feedback driving, the colloidal particles would start from an initial configuration gradually spreading out due to Brownian motion. During such a process, the entropy S_{B} of the water (bath) remains constant and the entropy of the bond lengths between the individual colloids (system) S increases. The diffusion of the center of mass of the colloidal molecule is insensitive to the feedback and the corresponding entropy always increases. To address the structure formation caused by the feedback, we thus consider the distribution of the bond lengths only. The total entropy production rate during the process reads $\dot{S}_{\mathrm{tot}} = \dot{S} > 0$. A similar time evolution is observed if one switches on the laser and targets random locations at the circumference of the particles, without using the information about the relative positions of the particles. In that case, a part of the laser power P a is absorbed by the particles and gradually dissipated to the bath, leading to $\dot{S}_{\mathrm{B}} = P_{\mathrm{a}}/T > 0$, where T is the bath temperature. The total entropy production rate during the process thus reads $\dot{S}_{\mathrm{tot}} = \dot{S} + \dot{S}_{\mathrm{B}} > 0$. In addition to that, the particles now spread faster than without the heating. Utilizing the information about particle positions according to the rules described in the main text in placing the laser implies a qualitatively different evolution of the system. The particles form localized structures which fluctuate due to the Brownian motion and oscillate due to the feedback. After long times, the system attains a non-equilibrium steady-state with a time-independent probability distribution of interparticle distances. The laser heating still produces nonzero entropy flux to the bath, but the system entropy is now time-independent. The total energy input into the system both in the situation without aiming the laser and with the aiming is the same, given by P_{a}. When comparing the random driving and the structure formed, the structure has a lower entropy than the random particle distribution which is due to the utilization of the information. It is the processing of this information which requires the additional energy input. More precisely, the information processing is necessarily accompanied with a positive entropy flux $\dot{S}_{\mathrm{F}}$, which is bounded from below by the Landauer's principle. The total entropy production rate in the steady-state thus reads $\dot{S}_{\mathrm{tot}} = \dot{S}_{\mathrm{B}} + \dot{S}_{\mathrm{F}} > 0$.

The feedback loop uses the information about the particle positions to balance the particle spreading due to the diffusion. Differently speaking, the feedback introduces a negative entropy influx $\dot{S}_{-}$ into the system. The described entropy fluxes through the system in the non-equilibrium steady-state are depicted in Supplementary Figure 6.

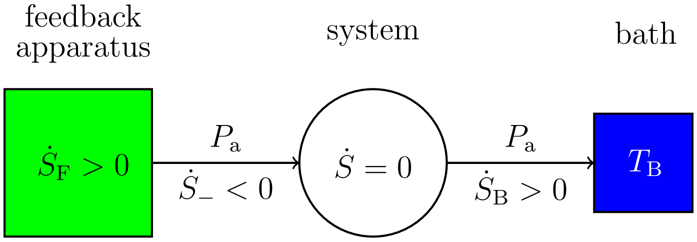

Supplementary Figure 6 | Thermodynamic diagram of the system.

In order to evaluate the negative entropy influx $\dot{S}_{-}$, it is possible to follow the analysis of reference[3] devoted to entropy production in stochastic systems with resetting. Let us for simplicity consider just the dimer case, which can be straightforwardly generalized to more particles. We denote as $\rho(x,t)$ the probability distribution for the bond length $x = r_{12}$ at time t. The rate of change of the Shannon entropy of this distribution reads

$$\dot{S} = -k_{\mathrm{B}} \int_{-\infty}^{\infty} \mathrm{d}x \ln \rho(x,t) \frac{\partial \rho(x,t)}{\partial t}. \tag{25}$$

Intuitively, this function is large after switching on the feedback in an unstructured system and decreases towards zero while the system relaxes to a time-independent non-equilibrium steady state, where $\partial \rho(x)/\partial t = 0$, $\rho(x) = \lim_{t\to\infty} \rho(x,t)$.

The dynamical equation for $\rho(x,t)$ can be written as

$$\frac{\partial \rho(x)}{\partial t} = 2D_0 \frac{\partial^2}{\partial x^2} \rho(x) + L[\rho(x,t)], \tag{26}$$

where the fist term stands for the change of the density due to the diffusion and the second one due to the driving. Inserting supplementary equation 26 into equation 25 we obtain for the entropy change in the steady-state

$$\dot{S} = -2D_0 k_B \int_{-\infty}^{\infty} dx \frac{\partial^2}{\partial x^2} \rho(x) \ln \rho(x) + S_{-} = 0. \tag{27}$$

Regardless the specific form of the operator $L[\rho]$ in supplementary equation 26, the negative entropy influx due to the feedback is determined by the stationary distribution $\rho(x)$:

$$\dot{S}_{-} = 2D_0 k_{\mathrm{B}} \int_{-\infty}^{\infty} \mathrm{d}x \ln \rho(x) \frac{\partial^2}{\partial x^2} \rho(x) = -2D_0 k_{\mathrm{B}} \int_{-\infty}^{\infty} \mathrm{d}x \frac{1}{\rho(x)} \left[\frac{\partial}{\partial x} \rho(x) \right]^2 < 0. \tag{28}$$

Let us now consider the case of vanishing time delay δt. Then the feedback creates the effective V-type potential $U(x) = \gamma v_{\mathrm{th}} \left| |x| - r_{\mathrm{eq}} \right|$ for the bond length, where $\gamma = 6\pi\eta R$ is the Stokes friction coefficient. The stationary distribution of the bond length thus reads $\rho(x) = \exp(-\gamma v_{\mathrm{th}} \left| |x| - r_{\mathrm{eq}} \right| / k_{\mathrm{B}} T)/Z$ and the formula (28) gives us

$$\dot{S}_{-} = -2D_0 k_{\mathrm{B}} \beta^2 \gamma^2 v_{\mathrm{th}}^2 = -2k_{\mathrm{B}} \beta \gamma v_{\mathrm{th}}^2 = -2\frac{\gamma v_{\mathrm{th}}^2}{T} = -2\eta \frac{P_{\mathrm{a}}}{T}. \tag{29}$$

Here ηP_{a} denotes the fraction of the absorbed light power (efficiency η, absorbed power P_{a}), which is used for the particle propulsion, $\beta = (k_{\mathrm{B}} T)^{-1}$ and Z as the equilibrium partition

function. Quite intuitively, the entropy influx is stronger (more negative) for larger velocities. Its absolute increase with decreasing temperature is not so intuitive. Considering a more general potential of the form kx^n we find that $\dot{S}_- \propto (k/T)^{2/n}T$. Thus the increase of $|\dot{S}_-|$ with decreasing T is slower for stronger potentials/more localized distributions and for $n \geq 2$ the absolute entropy influx even increases with T.

Supplementary Note 5: Experimental Setup

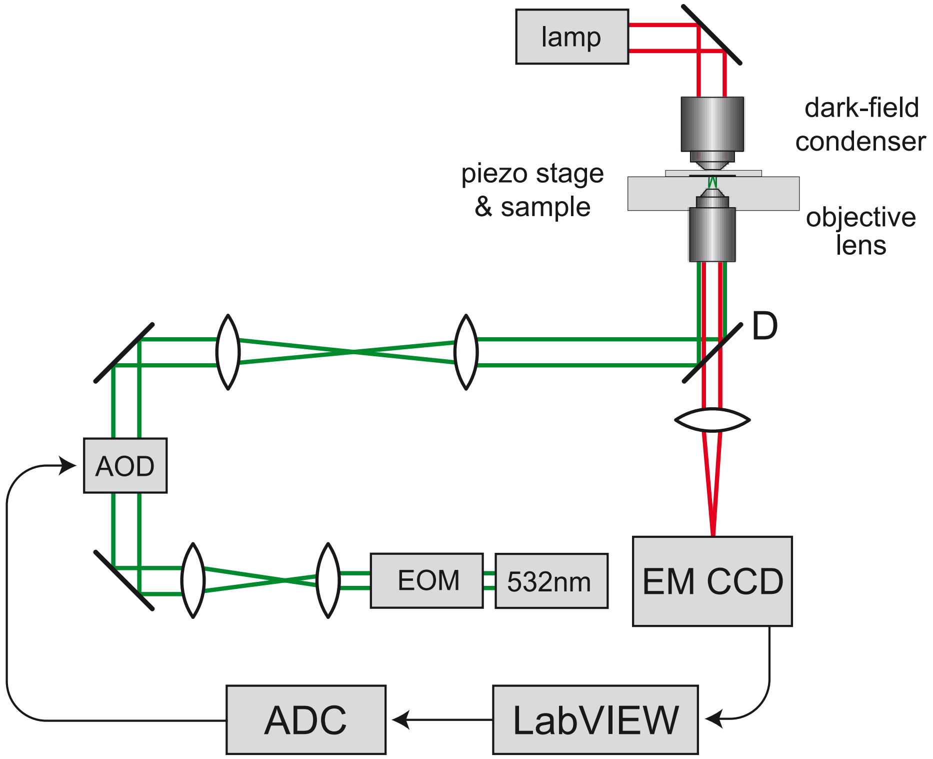

Supplementary Figure 7 | Experimental setup for the feedback controlled active particles. The following abbreviations are used: AOD – acousto-optic-deflector, EOM – electro-optical modulator, emCCD – Electron Multiplying CCD, FPGA – Field Programmable Gate Array, D – Dichroic mirror. All the other components are lenses, mirrors and a standard microscopy lamp.

Samples have been investigated in a custom built inverted microscopy setup. The setup is based on an Olympus IX 71 microscopy stand (see Supplementary Figure 7). Optical heating of the active particles is carried out by a CW 532 nm wavelength laser. The laser intensity is controlled by a Conoptics 350-50 electro-optical modulator (EOM). An acousto-optic deflector (AOD) together with a 4-f system (two f = 20 cm lenses) is used to steer the 532 nm wavelength laser focus in the sample plane. The AOD is controlled by a Field Programmable Gate Array (FPGA, National Instruments) via a LabView program. The calibration of the AOD for precise laser positioning is carried out using a 2D projection method developed in the lab A Leica 100x, infinity-corrected, NA 1.4 - 0.7 (set to 0.7), HCX

PL APO objective lens is used for focusing the 532 nm laser to the sample plane as well as for imaging the active particles. Active particles are imaged under dark field illumination. When the sample is placed under the microscope, an oil immersion dark field condenser (Olympus 1.2 NA) is approached from the top. The scattered light from the sample is collected with the Leica objective lens and imaged with a $f = 30$ cm tube lens to an emCCD camera (Cascade 650). A region of interest (ROI) of 200 pixels x 200 pixels is utilized for the real-time imaging, analysis and recording of the particles, with an exposure time of 80 ms or 110 ms.

Supplementary References

[1] Bregulla, A. P., Würger, A., Günther, K., Mertig, M. & Cichos, F. Thermo-osmotic flow in thin films. *Phys. Rev. Lett.* **116**, 188303 (2016).

[2] Bickel, T., Majee, A. & Würger, A. Flow pattern in the vicinity of self-propelling hot Janus particles. *Phys. Rev. E* **88**, 012301 (2013).

[3] Fuchs, J., Goldt, S. & Seifert, U. Stochastic thermodynamics of resetting. *Eur. Phys. Lett.* **113**, 60009 (2016).
