# Supplementary material for: Active particles bound by information flows
Source: Nat Commun. 2018 Sep 21;9:3864. doi: 10.1038/s41467-018-06445-1 (PMC6154969; doi:10.1038/s41467-018-06445-1)
Supplement: Supplementary file 3 — Description of Additional Supplementary files [file 41467_2018_6445_MOESM3_ESM.pdf]

The following movies display the raw images of the particles in the experiments. The images depicts a quadratic area of 200 pixels x 200 pixels, corresponding to 35 µm x 35 µm.

1. **Back and Forth Localization.mp4**: Single particle driven between two target positions with a heating power of 0.1 mW. The particle is trapped at the target position for 100 frames. Images are taken at an exposure time/inverse framerate of 80 ms.

2. **Six Particles Localization.mp4**: Localization of 6 particles at fixed positions along a ring with diameter 13.5 µm. The total used heating power is 1.25 mW. The radius is then increased, particles rotate as a whole on the ring to left and right. One of the particles is deployed to remote position from the ring and back integrated. A 7th and 8th particle is integrated later into the structure.

3. **Swarm and Pattern 8 Particles.mp4**: The movie shows the control of 8 particles. The total used heating power is 1.25 mW. All 8 particles are first moving between two target positions and are then localized at 8 positions along a ring with diameter 17.5 µm. The particles in this ring-like structure are rotated as a whole to the left and the right.

4. **Dimer.mp4**: Dimer structure of two active particles bound by the feedback rule mentioned in the text. The used laser power is 0.75 mW per particle and the adjusted bond length r_{eq} = 7.1 µm. The exposure time and inverse framerate is 110 ms.

5. **Trimer.mp4**: Trimer structure of three active particles bound by the feedback rule mentioned in the text. The used laser power is 0.5 mW per particle and the adjusted bond length r_{eq} = 7.1 µm. The exposure time and inverse framerate is 110 ms.

6. **Tetramer.mp4**: Tetramer structure of 4 active particles bound by the feedback rule mentioned in the text. The used laser power is 0.5 mW per particle and the adjusted bond length r_{eq} = 7.1 µm. At about 18 seconds the structure is isomerizing. The exposure time and inverse framerate is 110 s.

7. **Pentamer.mp4**: Pentamer structure of 5 active particles bound by the feedback rule mentioned in the text. The used laser power is 0.4 mW per particle and the adjusted bond length r_{eq} = 7.9 µm. The exposure time and inverse framerate is 110 ms.

8. **Hexamer.mp4**: Hexamer structure of 6 active particles bound by the feedback rule mentioned in the text. The used laser power is 0.4 mW and the adjusted bond length r_{eq} = 7.9 µm. The exposure time and inverse framerate is 110 ms.

9. **Dodecamer.mp4**: Dodecamer structure of 12 active particles bound by the feedback rule mentioned in the text. The used laser power is 0.2 mW per particle

and the adjusted bond length r_{eq} = 7.9 µm. 2 of the particles in the top left corner stick together. The exposure time and inverse framerate is 110 ms.
